# Female sex is not a risk factor for post procedural mortality in coronary bypass surgery in the elderly: A secondary analysis of the GOPCABE trial

**DOI:** 10.1371/journal.pone.0184038

**Published:** 2017-08-30

**Authors:** Gloria Faerber, Michael Zacher, Wilko Reents, Jochen Boergermann, Utz Kappert, Andreas Boening, Anno Diegeler, Torsten Doenst, for the GOPCABE investigators

**Affiliations:** 1 Department of Cardiothoracic Surgery, Jena University Hospital, Friedrich-Schiller-University of Jena, Jena, Germany; 2 Cardiovascular Clinic Bad Neustadt, Bad Neustadt, Germany; 3 Clinic for Heart, Thoracic and Cardiovascular Surgery, Heart and Diabetes Center NRW, Ruhr University Bochum, Bad Oeynhausen, Germany; 4 Department of Cardiac Surgery, Cardiovascular Institute, University of Dresden, Dresden, Germany; 5 Department of Cardiovascular Surgery, University of Gießen, Gießen, Germany; University of Adelaide, AUSTRALIA

## Abstract

**Objective:**

Female sex is considered a significant risk in cardiac surgery and is included in the majority of scores for risk assessment. However, the evidence is controversial and older women undergoing cardiac surgery have not specifically been investigated. We assessed the influence of female sex on surgical risk (30-day mortality) in a secondary analysis of the GOPCABE trial (German Off-Pump Coronary Artery Bypass grafting in the Elderly (GOPCABE) trial, comparing on- to off-pump) and also evaluated its impact on risk prediction from commonly used risk scores.

**Methods:**

We performed logistic regression analyses on the GOPCABE trial population, where patients were randomized to either on- or off-pump CABG. The study was performed in 12 cardiac surgery centers in Germany and analyzed 2394 patients having undergone CABG at age ≥75 years (1187 on-pump, 1207 off-pump). Of the 2394 patients, 755 (32%) were women. The logistic EuroSCORE and the German KCH score were calculated as expected (E) mortality and values were compared to observed (O) 30-day mortality (O/E ratio).

**Results:**

There was no difference in mortality or major cardiovascular adverse events after 30 days between men and women for both on- and off-pump CABG (men: on- vs. off-pump OR = 0.90, 95%-CI: [0.63;1.27]; women: on- vs. off-pump OR = 1.07, 95%-CI: [0.62;1.87]). Therefore, groups were combined for further analyses. Both men and women had considerable and similar comorbidities. Expected mortality was significantly higher for women than for men (logistic EuroSCORE: 8.88±6.71% vs. 7.99±6.69%, p = 0.003; KCH score: 4.42±3.97% vs. 3.57±3.65%, p = 0.001). However, observed mortality rates (O) tended to be even lower in women (2.1% vs. 3.0%). The O/E ratio was closer to 1 in men than in women (0.84 vs. 0.47). Excluding female sex from the risk models increased O/E ratio to 0.69.

**Conclusions:**

Female sex is not a risk factor in coronary bypass surgery in the GOPCABE population. The result is the same for on- and off-pump surgery. Since female sex is a component of most risk scores, the findings may identify a potential inaccuracy in current surgical risk assessment, specifically for elderly women.

**Trial registration:**

**Clinicaltrials.gov** GOPCABE trial No. NCT00719667

## Introduction

### Background

Sex differences in mortality have been intensively investigated, specifically in cardiac surgery [1–3]. For coronary artery bypass grafting (CABG) female sex has been included as risk factor in prevalent scores to assess perioperative risk, such as the EuroSCORE or the German coronary surgery (KCH) score [4, 5]. Thus, when quoting perioperative risk for patients with identical risk profiles, a man will receive a lower predicted mortality risk than a woman by most scores just because female sex as risk factor is included in the mathematical model.

However, several reports question the validity of female sex as an independent risk factor in CABG. They state that the described differences in mortality are not due to sex but due to different preoperative risk profiles women present at the time of surgery (e.g., more diabetes, hypertension and congestive heart failure, smaller bypass target vessels and therefore less complete revascularization or older age) [1, 3, 6–9]. However, these reports are limited by their retrospective nature being mainly database analyses. The GOPCABE trial provides a complete and prospective dataset from a randomized trial (comparing on- vs. off-pump CABG) [10]. The trial is unique in that it recruited two thirds of all patients having had bypass surgery at age above 74 at the participating centers within the recruitment period. It thereby also addresses an age group that has never been specifically investigated for sex differences.

Age is known as independent risk factor for mortality in cardiac surgery and finds application in commonly used risk scores (EuroSCORE, KCH score and STS score). It additionally seems to interact with patients’ risk profiles. With increasing patient age, risk profiles seem to change and intersex differences appear to decline [11, 12].

We therefore used the GOPCABE patient dataset, calculated two commonly used risk scores to predict perioperative mortality and assessed two key objectives 1. To assess the impact of female sex on outcome in coronary bypass surgery in patients above 74 years of age. 2. To estimate the accuracy of two commonly used risk scores (logistic EuroSCORE and the German KCH) to predict perioperative mortality in men and women. The results of the analysis will help to clarify a current controversy regarding risk assessment in elderly women undergoing coronary bypass surgery.

## Methods

### Study design

This secondary analysis used the dataset from the original GOPCABE trial population and specifically assessed the influence of female sex on perioperative outcome and the impact on predictive accuracy of commonly calculated risk scores (logistic EuroSCORE and the KCH score).

### Setting, participants and data sources

The GOPCABE trial is a randomized, controlled multicenter trial conducted at 12 German institutions that was aimed at evaluating the impact of on- versus off-pump coronary artery bypass grafting in the elderly [10]. The recruitment period was from June 25, 2008 to September 9, 2011. The study had an “all comers” design (i.e., all patients presenting for CABG surgery at age >74 were considered candidates for the trial) and randomized patients who were scheduled for isolated, first-time CABG at age ≥75, were either randomized to on- and off-pump CABG. The inclusion rate was 68.9% of all patients who underwent CABG at the participating centers during the recruitment phase. All patients were Caucasian. All patients received standard operative and postoperative care. Details on randomization and follow up are described in Diegeler et al. [10]. Patient data were obtained with pre-specified case report forms and entered into a central, internet-based, password protected database. All patients had provided written informed consent, which included further secondary analysis.

### Variables

The following variables were obtained as preoperative risk factors and found entry into the multivariable analyses: Age, Body Mass Index, diabetes mellitus, chronic obstructive pulmonary disease, peripheral vascular disease, cardiac rhythm, extent of coronary artery disease, history of myocardial infarction, history of percutaneous intervention, angina class, ejection fraction, stroke, renal function, center, on- / or off-pump surgery. The following variables were obtained as peri-operative characteristics: Duration of surgery and number of grafts, duration of mechanical ventilation, units of red blood cell transfusion, length of ICU and hospital stay.

We obtained 30-day mortality as primary variable and perioperative myocardial infarction, stroke, re-revascularization, renal replacement therapy, predicted mortality from logistic EuroSCORE and KCH score as secondary variables for the specific comparison of male vs. female sex. For the analysis of predicted risk for women without factor sex, the field for “female sex” was left blank when filling in the score calculation form.

### Study size

There were 2394 patients (755 women, 1639 men) for analysis of 30-day mortality.

### Statistical methods

Baseline and operative characteristics were compared with the use of the chi-square test, t-test or Kruskal-Wallis test, as appropriate. Dichotomous data are presented as numbers and percentages. Continuous data are presented as means and standard deviations or median and interquartile ranges. The continuity-corrected chi-square test was used for comparison of the 30-day end point. Treatment effects at 30 days are expressed as odds ratios and 95% confidence intervals. We also performed risk adjusted modelling and excluded the necessity to adjust for any of the known independent variables (see Table 1) in case sex should have an influence on the outcome variables. For logistic regression we used the conditional backward elimination. Since sex-specific comparisons of on- and off-pump surgery using logistic regression analyses did not reveal significant effects of either operative technique on mortality, groups were combined for further analyses. The EuroSCORE was calculated as described by Roques et al. [4] and the KCH score was calculated as described by the German Federal Quality Assessment System (BQS–Bundesgeschäftsstelle Qualitätssicherung gGmbH) [5]. For excluding “female sex” as a risk factor in the female population, the variable “female sex” was excluded from the algorithm [5] and the score was recalculated. All statistical analyses were performed with the SPSS software (IBM SPSS Statistics for Windows, Version 20.0. Armonk, NY: IBM Corp.).

**Table 1:** Baseline characteristics.

	Female	Male	p-value
	(n = 755)	(n = 1639)	
Age [years]	78.7 ± 2.99	78.5 ± 2.90	0.094
Body-mass-index [kg/m^2^]	28.4 ± 4.54	27.5 ± 3.78	<0.001
Insulin-dependent diabetes mellitus	147 (19.5%)	198 (12.1%)	<0.001
Chronic obstructive pulmonary disease	54 (7.2%)	191 (11.7%)	0.003
Pulmonary hypertension	20 (2.6%)	45 (2.7%)	0.367
Peripheral vascular disease	217 (28.7%)	563 (34.4%)	0.007
Sinus rhythm	678 (89.8%)	1393 (85.0%)	0.001
Extend of coronary artery disease			0.763
One-vessel disease	15 (2.0%)	22 (1.3%)	
Two-vessel disease	73 (9.7%)	152 (9.3%)	
Three-vessel disease	451 (59.7%)	991 (60.5%)	
Left main coronary artery disease (<75%)	7 (0.9%)	9 (0.5%)	
Left main coronary artery disease plus disease of one to three vessels	209 (27.7%)	465 (28.4%)	
History of myocardial infarction	255 (33.8%)	628 (38.3%)	0.036
History of percutaneous coronary intervention	159 (21.1%)	372 (22.7%)	0.397
CCS-angina class			0.005
I	47 (6.2%)	128 (7.8%)	
II	250 (33.1%)	571 (34.8%)	
III	343 (45.4%)	676 (41.2%)	
IV	43 (5.7%)	57 (3.5%)	
Left ventricular ejection fraction			<0.001
<30%	7 (0.9%)	57 (3.5%)	
30–50%	174 (23.0%)	532 (32.5%)	
>50%	574 (76.0%)	1050 (64.1%)	
Previous stroke	64 (8.5%)	152 (9.3%)	0.591
Creatinine level			0.022
≤ 2.3 mg/dl	742 (98.3%)	1577 (96.2%)	
> 2.3 mg/dl	8 (1.1%)	45 (2.7%)	
Renal replacement therapy	5 (0.7%)	17 (1.0%)	
Logistic EuroSCORE [%]	8.88 ± 6.71	7.99 ± 6.96	0.003
German KCH-score [%]	4.42 ± 3.98	3.57 ± 3.65	<0.001

Data are mean ± standard deviation or n (% of total). CCS = angina class according to the Canadian Cardiovascular Society. The two patient populations contain both, on- and off-pump population for each group.

## Results

### Participants

The 2394 included patients received either on- (n = 1207) or off-pump CABG (n = 1187). We had previously demonstrated the absence of overall differences between on- and off-pump in this patient population [10]. Comparing the sexes for on and off-pump surgery did also not reveal any significant differences. In men, the odds ratio (OR) for on-pump (n = 818) versus off-pump (n = 821) was 0.90 (95%-CI: [0.63; 1.27]). In women, the OR for on-pump (n = 389) versus off-pump (n = 366) was 1.07 (95%-CI: [0.62; 1.87]). For the following sex-specific comparison, we therefore combined the data for on- and off-pump patients.

### Descriptive data

Table 1 shows the preoperative and demographic data of the patient population separated by sex. Female patients had a higher body-mass-index and more insulin-dependent diabetes mellitus. In contrast, they had a lower incidence of chronic obstructive pulmonary disease and peripheral vascular disease, were more often in sinus rhythm, had less previous myocardial infarction, better left ventricular ejection fraction and lower creatinine levels. The extent of coronary artery disease did not significantly differ between the two groups and the remaining risk factors were relatively similar. The predicted risk of mortality as calculated with the logistic EuroSCORE and the KCH score were highest in women.

### Outcome data

Table 2 shows the perioperative characteristics of the two groups. There were no significant differences regarding duration of the operation, percentage of off-pump procedures, number of distal anastomoses, and length of mechanical ventilation, ICU- or hospital stay. Nevertheless, female patients received more red blood cell transfusions.

**Table 2:** Perioperative characteristics.

	Female	Male	p-value
	(n = 755)	(n = 1639)	
OP duration [min]	170 (142; 200)	170 (141; 201)	0.862
off-pump (%)	366 (48.5)	821 (51.1)	0.482
No of distal anastomoses	3 (2; 3)	3 (2; 3)	0.343
Duration of mechanical ventilation [h]	12 (8; 16)	12 (9; 16)	0.636
Units of red blood cell transfusions	2 (0; 3)	1 (0; 3)	<0.001
Postoperative length of ICU stay [days]	2 (1; 4)	2 (1; 4)	0.509
Postoperative length of hospital stay [days]	9 (8; 12)	9 (8; 12)	0.240

Values are median (25th and 75th percentile) or n (%). OP: Operative Procedure, ICU: Intensive care unit

### Main results

Table 3 shows a sex-specific analysis of the primary (death) and secondary variables (myocardial infarction, repeat revascularization, stroke, or new renal replacement therapy). There was no increased risk of death or rate of complications associated with female sex. In contrast, there was a trend towards lower odds-ratios for female sex, but the difference was not significant.

**Table 3:** Sex-specific analysis of primary and secondary variables.

End point (at 30 days)	Female	Male	Odds Ratio	p-value
			[95% CI]	
*Primary variable*:				
Death	16 (2.1%)	49 (3.0%)	0.703 [0.397; 1.244]	0.279
*Secondary variables*:				
Myocardial infarction	7 (0.9%)	31 (1.9%)	0.485 [0.213; 1.107]	0.111
Repeat revascularization	3 (0.4%)	17 (1.0%)	0.381 [0.111; 1.303]	0.147
Stroke	22 (2.9%)	36 (2.2%)	1.226 [0.781; 2.288]	0.317
New renal-replacement therapy	16 (2.1%)	50 (3.1%)	0.688 [0.389; 1.216]	0.227
*Combined endpoint*	50 (6.6%)	142 (8.7%)	0.748 [0.535; 1.045]	0.090

Values are n (% of total population) or ratios, CI = confidence interval, based on intention to treat analysis. Odds ratios are reported for end points at 30 days after surgery.

Table 4 shows the comparison of predicted mortality rates estimated by the logistic EuroSCORE or the German KCH-score with the observed mortality rates. For men, the logistic EuroSCORE indicates an expected mortality of approximately 8%. For the same patients, expected mortality based on the KCH-score was less than half, pointing towards the known overestimation of mortality by the EuroSCORE. The observed mortality rate for these men was 3% at 30 days. For women, both scores expected higher rates than for men. When female sex as a risk factor was excluded from the calculation, expected mortality rates for women were significantly lower. The observed mortality rate for women was 2.1% at 30 days.

**Table 4:** Observed and predicted perioperative mortality according to sex.

	Female		Male
	With factor sex	Without factor sex	
Mortality rates at 30-days [%]	2.1	2.1	3.0
Logistic EuroSCORE [%]	8.88 ± 6.71	6.38 ± 4.82*	7.99 ± 6.96
German KCH-score [%]	4.42 ± 3.98	3.05 ± 2.89*	3.57 ± 3.65

Score values are mean ± SD

* p<0.05 versus “with factor sex”

**Fig 1:**
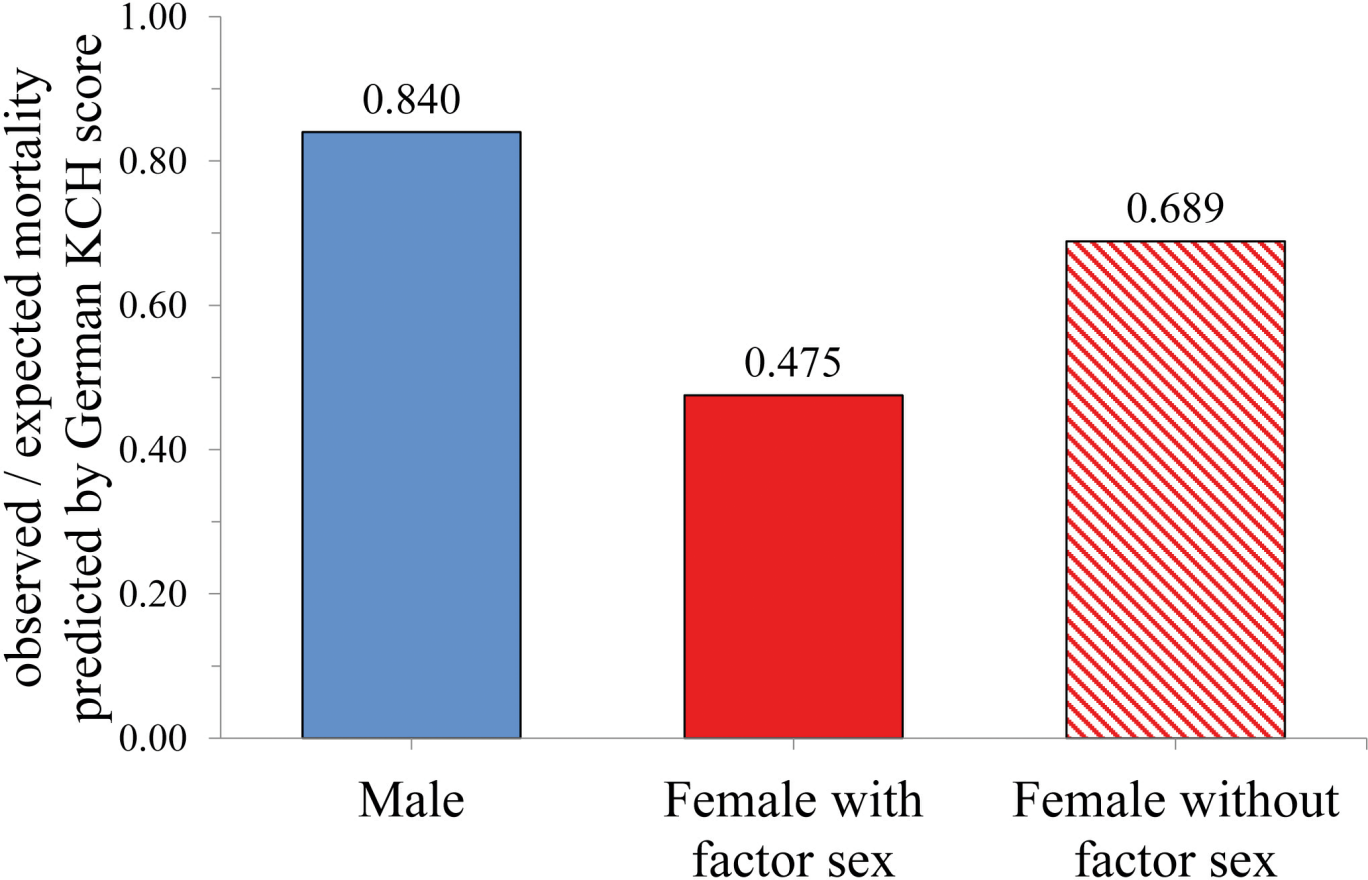
Relationship of observed to expected mortality (O/E ratio) predicted by the German KCH score for the GOPCABE trial population. Relationship of observed to expected mortality (O/E ratio) predicted by the German KCH score for male and female patients of the GOPCABE trial population. Risk for women was analyzed either with or without factor sex. Note that eliminating factor sex from the risk model substantially increased O/E ratio (see methods for details).

Fig 1 shows the relationship of observed to expected mortality (O/E ratio) for men and women predicted by the KCH-score. For men, the O/E ratio was 0.84 suggesting a slight overestimation of true mortality by the score. In women, this ratio was only 0.48 indicating a much stronger overestimation of mortality. Calculating KCH-scores for women without “female sex” as risk factor corrects the strong overestimation resulting in an O/E ratio of 0.69.

## Discussion

### Key results

We demonstrate in this secondary analysis of the GOPCABE trial that female sex does not appear to be a risk factor for 30-day mortality in coronary bypass surgery in the GOPCABE-population and that the result is not related to the use of cardiopulmonary bypass. Since female sex is a component of most risk scores, the findings may identify a potential inaccuracy in current surgical risk assessment, specifically for elderly women.

### Interpretation

Whether female sex is a risk factor in cardiac surgery has been a discussion for decades [6, 13–21]. We demonstrate in this secondary analysis of the GOPCABE trial that female sex does not increase mortality in CABG patients. This finding is underscored by several other investigators who also failed to identify an influence of female sex on mortality, specifically in patients undergoing aortic valve surgery [22, 23] or tricuspid valve [24, 25] surgery. Since female sex is included in most risk scores (e.g., EuroSCORE, STS score, German KCH-score, German aortic valve score), we illustrate here the impact of this inclusion on the ability to predict true mortality rates. The example in Fig 1 shows that risk score calculation substantially overestimates true risk, an error that is corrected if the factor “female sex” is excluded from calculation. This finding is supported by several other studies that also found that female sex is not associated with increased mortality [6, 14, 26]. Some studies even suggested a protective effect of female sex on long term survival after CABG [1, 27]. Consistently with the last two opinions, in the GOPCABE study population, female sex was not a risk factor for increased mortality. Mortality rate for women even tended to be lower than for men (2.1 vs. 3%), an observation that was consistent up to one year.

Thus, our results and those of others [1, 6, 14, 26, 27] suggest that the impact of female sex as a risk factor in coronary artery bypass surgery should be re-evaluated and current risk scores should be readjusted specifically for this older patient population.

One discussed explanation for the published differences in outcome has been a difference in sex-specific comorbidities. Several studies have shown that women and men do not share all the same predictors of mortality after surgery [3, 6, 9]. Main predictors in female patients were older age, diabetes, hypertension, congestive heart failure, unstable angina and more often urgent revascularization [9, 12, 17, 28]. Male patients presented more often with an ejection fraction of less than 35%, triple vessel disease, repeat operations and recent history of smoking [29]. We made the same observations within the GOPCABE population. On the one hand, female patients had a higher body-mass-index and more insulin-dependent diabetes mellitus. On the other hand, they had a lower incidence of chronic obstructive pulmonary disease and peripheral vascular disease, were more often in sinus rhythm, had less previous myocardial infarction, better left ventricular ejection fraction and lower creatinine levels. However, despite the differences in comorbidity profiles, women and men had similar outcomes.

Another possible explanation for differences in outcome could be related to the different age at which women and men undergo surgery. In many studies, women were significantly older than men [1, 9, 16, 27], suggesting that age but not sex may be the key risk factor for mortality. Koch et al. [7] showed in a propensity-matched analysis comparing men and women of the same age (65 years), that female sex was not associated with increased mortality. However, only 26% of women included in that study could be matched with men properly. This difficulty in matching men and women has led to the conclusion that a selection bias can never be completely eliminated in studies comparing treatment outcomes between men and women. With respect to age, we support the findings by Koch et al. [7]. In GOPCABE, women and men were the same age (78 ± 3 years) and outcome was also the same.

Since risk profiles change with age, it is possible that our results are not representative for the younger population. Female patients may indeed have a higher mortality risk if they need CABG at younger age. Below 50 years of age, mortality risk for women has been reported to be up to 3 times higher compared to male patients [12, 17, 30]. With increasing age those intersex differences seem to decline [11, 12]. As the main inclusion criterion for the GOPCABE trial was age above 74 years, it is reasonable to speculate that potential sex-specific effects on mortality that may be present at younger age are no longer detectable in the elderly. However, it is important to realize in this context that most scores include sex as age-independent risk factor.

We used the EuroSCORE and the German KCH score (score used for operative risk assessment based on the German national database, similar to the STS score) because those two scores were used for risk assessment in Germany at the time of patient inclusion into the GOPCABE trial. Although the logistic EuroSCORE is no longer recommended by the Guidelines [31] and the KCH score is not internationally used, they serve well to illustrate the principle of one of our main conclusions, i. e. that current scores may inaccurately predict risk for women. For current international scores, female sex increases relative risk by 20% in the STS score and by 25% in the EuroSCORE II [32, 33]. Thus, this problem is still affecting risk prediction in our current daily practice.

Besides the preoperative characteristics, intraoperative variables such as surgical technique and graft selection with respect to female sex have been suggested to affect outcome [34, 35]. It has been discussed that women may present with smaller target vessels, increasing the technical challenge during CABG [8]. Some reports state that female patients receive incomplete revascularization more often [30, 36]. Furthermore, female patients seem to receive fewer arterial grafts [1, 8, 19, 37]. As it has been demonstrated that arterial grafts may have higher patency rates than saphenous vein grafts [38] and that reduced use of the IMA or radial artery may correlate with increased morbidity and mortality [1, 8, 15, 39–42], these technical aspects may mediate sex-specific differences in outcome. In the GOPCABE trial, there were no differences in the extent of coronary artery disease, number of distal anastomoses and the use of arterial grafts between women and men. Additionally, 95% of the patients received an IMA-graft. Thus, the data appear very valuable for our analysis. Nevertheless, a selection bias can never fully be excluded and one possibility for the lack of differences in our analysis may be the selection of very experienced surgeons for the trial patients raising the detection threshold for differences.

### Limitations

In addition to the above discussions and interpretations, several limitations apply to our study. First, another type of selection bias applies because the dataset is based on a prospective, randomized multicentre trial. Since most trials only recruit a small percentage of the patients treated in daily routine, the findings may not be representative, specifically if male-female comparisons are performed in this preselected patient population. However, the GOPCABE trial was a true “all comers” study with a high patient inclusion rate of 68.9% of all patients treated in the participating centers for the disease and age spectrum addressed by the trial. Therefore, resulting data should be representative and applicable to surgeons’ daily practice. Yet another selection bias addresses the selection of senior surgeons for trials. However, the impact of this selection should equally affect men and women. Second, all patients were Caucasian and the results may not be representative for other ethnic groups. Third, we did not perform propensity matching. However, given the complete lack of differences identified by multivariable analyses make the detection of significant differences by adding a propensity matching strategy highly unlikely.

### Generalisability

To the best of our knowledge, this is the only analysis addressing the influence of female sex on perioperative mortality specifically in the elderly population. We demonstrate in this secondary analysis of the GOPCABE trial that female sex is not a risk factor for 30-day mortality in coronary bypass surgery in the GOPCABE-population and that the result is not related to the use of cardiopulmonary bypass. Since female sex is a component of most risk scores, the findings may identify a potential inaccuracy in current surgical risk assessment, specifically for elderly women.

One consequence of the above discussions may be the suggestion to create a special risk score for older patients. One may even consider including male sex in such an age-dependent score. In any case, current risk assessment is flawed and patient information for women appears biased if recommendations are given based on mortality predictions from current risk scores.
